# Intermanual transfer and bilateral cortical plasticity is maintained in older adults after skilled motor training with simple and complex tasks

**DOI:** 10.3389/fnagi.2015.00073

**Published:** 2015-05-07

**Authors:** Daina S. E. Dickins, Martin V. Sale, Marc R. Kamke

**Affiliations:** Queensland Brain Institute, The University of Queensland, St LuciaQLD, Australia

**Keywords:** motor training, aging, transcranial magnetic stimulation (TMS), plasticity, intermanual transfer

## Abstract

Intermanual transfer refers to the phenomenon whereby unilateral motor training induces performance gains in both the trained limb and in the opposite, untrained limb. Evidence indicates that intermanual transfer is attenuated in older adults following training on a simple ballistic movement task, but not after training on a complex task. This study investigated whether differences in plasticity in bilateral motor cortices underlie these differential intermanual transfer effects in older adults. Twenty young (<35 years-old) and older adults (>65 years) trained on a simple (repeated ballistic thumb abduction) and complex (sequential finger-thumb opposition) task in separate sessions. Behavioral performance was used to quantify intermanual transfer between the dominant (trained) and non-dominant (untrained) hands. The amplitude of motor-evoked potentials induced by single pulse transcranial magnetic stimulation was used to investigate excitability changes in bilateral motor cortices. Contrary to predictions, both age groups exhibited performance improvements in both hands after unilateral skilled motor training with simple and complex tasks. These performance gains were accompanied by bilateral increases in cortical excitability in both groups for the simple but not the complex task. The findings suggest that advancing age does not necessarily influence the capacity for intermanual transfer after training with the dominant hand.

## Introduction

Neuroplasticity refers to the ability of the brain to alter its structure and function, allowing for adaptation to changing demands of the environment. Such neural flexibility plays a fundamental role in the formation and storage of memories, in learning new skills and behaviors, and in recovery of function following brain injury. Evidence suggests that, compared to younger adults, plasticity is reduced in older individuals. For example, studies inducing plasticity experimentally by motor training or using non-invasive brain stimulation have shown that plasticity is reduced in the motor cortex of older adults (Sawaki et al., 2003; Müller-Dahlhaus et al., 2008; Rogasch et al., 2009; Fathi et al., 2010; Todd et al., 2010). It has also been shown, however, that older adults frequently exhibit more diffuse neural activity, both within and between hemispheres, than do younger adults when performing the same task (Heuninckx et al., 2005). Thus, plasticity supporting a given function may manifest differently across neural networks in young and older individuals. The aim of the current study was to investigate whether the distribution of plasticity is altered in older compared to younger adults and to determine if any change in plasticity impacts the transfer of the learned motor skill to the untrained hand.

Advancing age is associated with great functional change in multiple neural systems (see Seidler et al., 2010 for review). In the motor system, aging is associated with a reduction in the specificity of neural activity during the performance of motor tasks. Specifically, when performing the same task older adults demonstrate greater activity across the hemispheres than do young adults (Calautti et al., 2001; Hutchinson, 2002; Mattay et al., 2002; Ward and Frackowiak, 2003; Heuninckx et al., 2005; Naccarato et al., 2006; Carp et al., 2011; Inuggi et al., 2011). It has been proposed that such bilateral over-activation may reflect a reduction in the ability of the aged brain to regulate activity in specific motor networks, which can disrupt normal brain function and lead to a decline in motor performance (Li and Lindenberger, 1999; Riecker et al., 2006; Langan et al., 2010; Bernard and Seidler, 2011; Inuggi et al., 2011). Alternatively, over-activation may reflect a compensatory mechanism, whereby additional brain regions are recruited to compensate for the effects of age-related neurobiological change, thereby assisting in maintaining motor function (Naccarato et al., 2006; Heuninckx et al., 2008; Goble et al., 2010; Kim et al., 2010). In either case, evidence of over-activation suggests that plasticity induced by motor-skill training may manifest more diffusely across bilateral motor cortices in older relative to younger adults. Moreover, activity (and hence plasticity) in both hemispheres may be necessary to support learning of a unilateral motor skill in older adults.

Few studies have compared bilateral plasticity in M1 of young and older adults following training on a unilateral motor task. Evidence from studies using transcranial magnetic stimulation (TMS) to probe plasticity, however, suggests that a bilateral change in cortical excitability is not a characteristic unique to the older brain. For example, there is a wealth of studies investigating the process whereby a motor skill transfers from a trained to an untrained hand, which is termed intermanual transfer (van Mier and Petersen, 2006; Koeneke et al., 2009; Stöckel and Wang, 2011; Pan and Van Gemmert, 2013; Parikh and Cole, 2013). It has been shown that practicing a simple motor task (ballistic finger abductions) is associated with intermanual transfer in young adults, and that this transfer is dependent on bilateral increases in cortical excitability (Carroll et al., 2008; Lee et al., 2010; Parikh and Cole, 2013). Specifically, Lee et al. (2010) demonstrated that in young adults disrupting cortical plasticity, by applying repetitive TMS to either the left or right motor cortices immediately following training, reduced training-related gains in motor performance for the contralateral hand. This finding suggests that bilateral plasticity induced in the trained and untrained hemispheres in young adults specifically supports performance gains of the trained and untrained hand, respectively. Interestingly, intermanual transfer of a simple ballistic motor task has been reported to be reduced (Parikh and Cole, 2013) or even absent (Hinder et al., 2011) in older adults, but changes in cortical excitability in both the trained and the untrained hemispheres are comparable to that found in the young (Hinder et al., 2011). The reduction or absence of intermanual transfer in the presence of increases in bilateral cortical excitability suggests that plasticity within both the trained and untrained hemisphere of older adults is necessary to support learning with the trained hand.

Although intermanual transfer of learned tasks has been shown to be reduced in older adults, this effect appears to depend on the type of task employed. Parikh and Cole (2013) reported that older adults who demonstrated reduced intermanual transfer following training on a simple task nonetheless exhibited comparable transfer to young adults after training on a complex grip and lift task. In contrast, task complexity does not seem a determining factor with young adults, who show transfer following training on ballistic finger abductions (Carroll et al., 2008), maze tracing (van Mier and Petersen, 2006), dexterity and precision tasks (Pereira et al., 2011), visuomotor adaptation (Pan and Van Gemmert, 2013), and object weight adaptation (Parikh and Cole, 2013). It is well established that undertaking simple and complex tasks draws upon differential neural circuitry (Sadato et al., 1996; Mima et al., 1999; Ehrsson et al., 2000, 2001; Solodkin et al., 2001; Verstynen et al., 2005; Davare et al., 2010; Holmström et al., 2011; Parikh and Cole, 2013). For example, it has been demonstrated that, in young adults, activity is more widespread when performing a sequence of movements with multiple fingers than when performing simple repetitive tapping movements with a single finger (Verstynen et al., 2005). The same is apparent when young adults perform skilled object manipulation compared to single-joint finger movements (Sadato et al., 1996; Mima et al., 1999; Ehrsson et al., 2000, 2001; Davare et al., 2010; Holmström et al., 2011). Similarly, although bilateral changes in cortical excitability following training on complex tasks have not been investigated in older adults, data from young adults suggests that regions outside the ipsilateral motor cortex might mediate transfer of complex tasks (see Tanji, 2001 for review). Indeed, when cortical excitability was assessed in bilateral motor cortices after training on a complex task, changes in cortical excitability were limited to the trained M1 (Pascual-Leone et al., 1995). Thus, because complex tasks recruit a more extensive and widespread network, which does not rely on the untrained motor cortex to facilitate learning of the untrained hand, age-related over-activation in the untrained motor cortex may not interfere with intermanual transfer of complex tasks. The influence of task complexity on intermanual transfer in young and older adults, however, has not yet been investigated in conjunction with measures of bilateral cortical excitability.

The current study aimed to investigate whether intermanual transfer and bilateral cortical excitability are altered in young and older adults after motor training on simple and complex tasks. In separate sessions, young and older participants trained on a simple (repeated ballistic thumb abduction) and a complex (finger-to-thumb opposition) task with the dominant hand. It was hypothesized that plasticity in both motor cortices is required for unilateral motor learning in older but not young adults. Thus, whilst both groups should show bilateral increases in cortical excitability following training on a simple ballistic task, it was predicted that intermanual transfer would be reduced in the older adults. With the complex task, however, it was predicted that intermanual transfer would be maintained in older adults. Based on the notion that intermanual transfer of a complex task is supported by more widespread neural activity, predominantly outside the primary motor cortices, it was predicted that any increase in M1 excitability after training would be limited to the trained hemisphere in young adults. In contrast, plasticity may still manifest bilaterally in older adults to support the motoric component of learning a complex task in the trained hand.

## Materials and Methods

### Participants

A total of 20 young participants between the ages of 18 and 33 years (*M* = 24.25, SD = 4.60, Males = 10) and 20 older participants between the ages of 65 and 77 years (*M* = 70.00, SD = 3.42, Males = 10) were tested. According to the Edinburgh handedness inventory (Oldfield, 1971) all but one young participant, who was classed as ambidextrous, were right-handed (Young *M* = 82.92, SD = 22.66, Range = 33.33–100; Older *M* = 83.30, SD = 16.13, Range = 44.44–100). Participants were recruited by word of mouth and through advertising in online newsletters and were reimbursed $10 per hour for their participation. Prior to commencement of testing all participants completed a TMS safety-screening questionnaire (Rossi et al., 2009, 2011) and provided fully informed written consent. All procedures were approved by The University of Queensland Medical Research Ethics Committee. Individuals with neurological disease or damage, epilepsy, history of head injury or psychiatric disorder, or who were taking neuroactive medications were excluded from the study. All participants had normal or corrected to normal visual acuity. There were no adverse reactions to the TMS.

### Transcranial Magnetic Stimulation

Transcranial magnetic stimulation was administered using a figure-of-eight shaped coil with a wing diameter of 70 mm, connected to a Magstim 200^2^ stimulator (Magstim Co., UK). TMS was delivered to the motor hotspot, which was defined as the optimal position on the scalp for evoking the largest and most consistent motor-evoked potential (MEP; peak-to-peak amplitude) in the target muscle, the *abductor pollicis brevis* (APB) of the left and right hands. Motor hotspots were located by placing the coil tangentially on the scalp with the handle pointing toward the back of the head, angled 45° from the midline and moving it systematically in a grid-like pattern. Stimulation occurred approximately every 5 s at an intensity sufficient to evoke a clear MEP in the target muscle. The position of the coil for each hotspot was recorded using a frameless infrared stereotaxic neuronavigation system (Visor, ANT, Netherlands). This navigation system was used to reproduce coil angle and location within an experimental session.

Following determination of the hotspot, resting motor threshold (rMT) was obtained for the cortical representation controlling the left and right APB. The rMT was defined as the minimum TMS intensity that evoked an MEP of above 50 μV in at least three out of five consecutive trials. The intensity of the TMS was adjusted using a staircase (two-down, one-up) procedure until the criterion was met. Following this, TMS test intensities were established for the left and right APBs. The test intensity was defined as that required to evoke an average MEP of approximately 1 mV (range 0.5 and 1.5 mV peak-to-peak) in the resting muscles. Average MEP amplitude at baseline and post-training was determined using the test intensity from a block of 21 TMS pulses that were delivered every 5 ± 1 s.

### Recording of Muscle Activity

Activity from the muscles of interest was recorded using surface electromyography (EMG). Disposable 24 mm silver–silver chloride (Ag/AgCl) electrodes were used, with the active electrode placed on the belly of the APB muscle of the left and right hands and reference electrodes on the metacarpophalangeal joint of the respective thumb. MEP data were amplified (x1000), filtered (20–1000 Hz) and sampled at 2000 Hz using a NeuroLog system (Digitimer, UK). Individual sweeps were sampled from 500 ms before stimulation to 500 ms after stimulation and stored for off-line analysis using Signal software (CED, UK). Muscle activity was visually monitored throughout the experiment using a digital oscilloscope. If activity occurred during a trial, participants were verbally prompted to relax. Acceleration data was amplified (x10), low pass filtered (x1000) and sampled at 2000 Hz. Consistent with Hinder et al. (2011, 2013), each sweep was triggered when the abduction acceleration exceeded approximately 4.9 m/s^2^. This threshold was reduced for participants exhibiting acceleration below this point.

### Simple Task: Repeated Ballistic Thumb Abduction

For the simple task condition participants were required to perform a volitional ballistic thumb abduction movement. The participants’ arms and hands were placed on cushioned platforms on the desk with the forearm supinated. The fingers and wrist of both hands were immobilized by straps attached to the platforms. Participants were instructed to move only the thumb as quickly as possible across the hand in a horizontal plane with the aim of maximizing peak acceleration.

Prior to, and at two and 15 min following training, participants performed this movement for a period of 30 s (15 trials at 0.5 Hz), with each individual movement initiated in response to an auditory tone. Each participant was given only one practice of the movement on each hand before baseline performance was recorded. Measures of performance in the right hand always preceded the left hand, pre and post-training. Performance on the simple task was quantified using an accelerometer attached to the right and left thumb. At each time point performance was defined as the peak acceleration averaged across 15 trials for each hand. Verbal encouragement, instructing participants to maximize their peak acceleration, was given prior to each block of the pre and post-measures. Visual feedback (see Simple Task Training Intervention) and verbal encouragement were given during training but not during the pre and post-measures.

#### Simple Task Training Intervention

During training, participants performed the same ballistic movement with the right hand paced to the auditory tone (0.5 Hz) for 1 min, followed by a 30 s rest period. This procedure was repeated ten times, resulting in a total of 10 min of training (300 ballistic movements). The accelerometer was fixed to the right thumb and was recording throughout the training intervention. Participants were provided visual feedback regarding their peak acceleration and were verbally encouraged to maximize peak acceleration throughout the training blocks.

### Complex Task: Finger-to-Thumb Opposition

**FIGURE 1:**
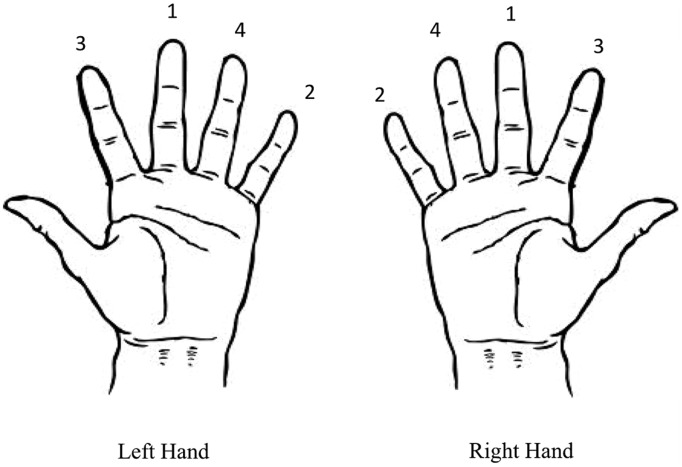
**Finger-to-thumb opposition sequence for the left and right hands.** Participants were instructed to move the thumb toward each of the fingers sequentially in the sequence depicted.

In the complex condition participants were required to perform a finger-to-thumb opposition task with the right hand first, followed by the left. This paradigm was modified from that of Karni et al. (1995), which induced robust changes in M1 activity following training. The sequence of movements is depicted in **Figure 1**. Prior to, and at two and 15 min post-training, participants were instructed to perform this sequence as quickly and as accurately as possible to maximize the number of sequences completed in a 30 s time period. Performance in each hand was quantified by determining the number of sequences performed correctly in the allotted time period (30 s). Performance was recorded using a digital video camera and stored for oﬄine analysis. Participants completed four practice sequences before baseline measures were initiated.

#### Complex Task Training Intervention

During training on the complex task participants performed the same sequence as in the pre- and post-training measures, but for training the timing of each individual finger-thumb opposition movement was paced by the same auditory cue used in the simple task. That is, each movement in the sequence depicted in **Figure 1** was performed at a rate of 0.5 Hz. As for the simple task, participants completed 10 blocks of training lasting 1 min each. These blocks were separated by a 30 s rest period. Performance was recorded throughout the training intervention using a digital video camera.

### Experiment Design and Procedure

**FIGURE 2:**
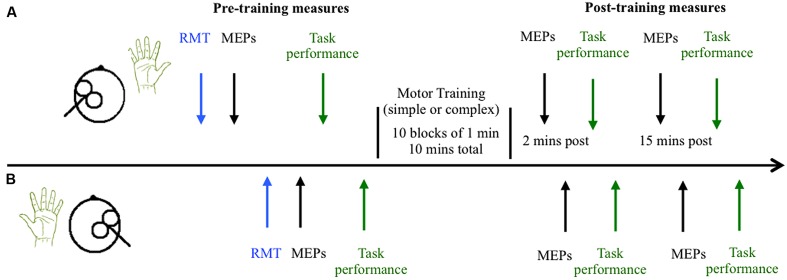
**Time course of experiment.** The timeline of measures is shown for the left hemisphere/right hand (trained M1 and hand) in **(A)** and the untrained right hemisphere/left hand in **(B)**. Cortical excitability was quantified by measuring the amplitude of motor evoked potentials (MEPs) elicited by 21 single pulses of transcranial magnetic stimulation in the resting right and left abductor pollicis brevis (APB) muscles before and after training. Behavioral performance was also quantified before and after training.

Participants completed two sessions at similar times of day at least 48 h apart, with each session lasting up to 2 h. During the training sessions participants were seated comfortably with both their right and left arms resting on cushioned platforms on a desk. The skin of both hands was cleaned thoroughly to minimize skin impedance and the surface electrodes were placed in position. The time course of the experiment is outlined in **Figure 2**. Participants received single pulse TMS to left and right M1 to locate the motor hotspot and to quantify cortical excitability before the motor training task. The task was then explained to the participants, and they were provided a brief chance to practice. Baseline measures of behavioral performance were then obtained from the right and left hands, followed by completion of the training task. The order of the simple and complex sessions was randomized across groups and individuals. Cortical excitability was assessed with single pulse TMS 2 and 15 min following training. MEPs were obtained from the left M1 (target hemisphere) and then the right M1 (non-target hemisphere) using the test stimulus intensity. Following assessment of cortical excitability at each time point, behavioral performance was re-measured. An eye tracker was used throughout the pre and post-measures to ensure participants kept their eyes open during the experiment.

### Data Processing and Analyses

The first trial from each block of MEP data was removed and the remaining trials in each block were averaged for each participant (20 MEPs per block). Trials containing voluntary muscle activity in the 100 ms before TMS were also removed, these constituted 2.71% of all remaining trials. Baseline rMTs were analyzed using a 2 × 2 ANOVA with the between-subjects factor age (young, older) and the within-subjects factor hemisphere (trained, untrained). Mixed ANOVAs were carried out for the behavioral data with the factors of time (pre, 2 min post, 15 min post), hand (trained and untrained) and age (young, older). For the simple task the dependent variable was the average maximum peak acceleration, whereas for the complex task it was the average number of sequences completed correctly in the allotted time (30 s). The effect of training on excitability of the APB muscles was analyzed using a 2 × 2 × 3 mixed ANOVA with the between-subject factor of age (young, older) and within-subject factors muscle (rAPB, lAPB) and time (pre, 2 min post, 15 min post). Separate ANOVAs were conducted for the simple and complex tasks. Bonferroni corrections were applied to all follow-up, two-tailed *t*-tests. To further explore the influence of plasticity (cortical excitability) on intermanual transfer a transfer index was calculated, which reflected the performance gain in the untrained hand expressed as a percentage of the gain in the trained hand. The relationship between the transfer index and MEP change in the untrained hemisphere was assessed using Pearson’s correlation analyses. Prior to analysis the data were examined for outliers and points of high influence using studentized deleted residuals, centered leverage values, and Cook’s distance. Separate analyses were conducted for young and older adults.

## Results

### Behavioral Performance in the Simple Task

**FIGURE 3:**
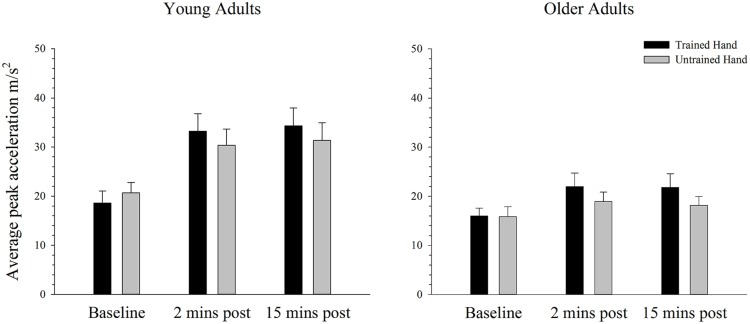
**Mean peak acceleration before and after training on a simple motor task in young and older adults.** Average peak acceleration increased significantly from pre to 2 and 15 min post-training in both young and old adults (*p* < 0.05). The increase was significantly greater in young adults and in the trained hand, but there was no difference between the groups in the transfer of the training effects to the untrained hand. Error bars denote SEM.

**Figure 3** depicts average peak acceleration in the trained and untrained hands for young and older adults at each time point before and following training. As can be seen in **Figure 3**, there was an increase in performance in both groups following training, but this effect is larger for the young than the older adults. ANOVA confirmed a training-related effect with a significant main effect of time; *F*(2,76) = 37.41, *p* < 0.001, $\eta_{p}^{2}$ = 0.50. Moreover, ANOVA also confirmed that the training-related increase in performance varied between young and older adults with a significant interaction between time and age; *F*(2,76) = 9.32, *p* = 0.003, $\eta_{p}^{2}$ = 0.20. Independent samples *t*-tests, which compared peak acceleration between each of the three time points in young and old adults revealed that in young adults peak acceleration increased significantly from baseline to the two [*t*(19) = 5.89, *p* < 0.001] and 15 min [*t*(19) = 5.32, *p* < 0.001] post-training measures. Older adults also demonstrated significantly greater peak acceleration relative to baseline at the two [*t*(19) = 3.22, *p* < 0.004] and 15 min post-training time points [*t*(19) = 2.78, *p* = 0.012], but the significant interaction indicates that the training-related effects were smaller in this group. There was no significant difference in peak acceleration between the two post-training measures in young [*t*(19) = 1.29, *p* = 0.214] or older adults [*t*(19) = 0.98, *p* = 0.338]. **Figure 3** also demonstrates that peak acceleration increased to a greater degree in the trained hand than in the untrained hand, irrespective of the participant’s age. ANOVA revealed that the effect of training varied between the hands with a significant hand × time interaction; *F*(2,76) = 10.08, *p* = 0.001, $\eta_{p}^{2}$ = 0.21. Paired samples *t*-tests demonstrated that while peak acceleration did not differ between the hands at baseline [*t*(39) = 1.56, *p* = 0.128], peak acceleration was significantly greater in the trained hand at both the two [*t*(39) = 3.35, *p* = 0.002] and 15 min [*t*(39) = 3.07, *p* = 0.004] post-training time points. There were no other significant main effects or interactions (all *p’s* > 0.436).

### Changes in Cortical Excitability in the Simple Task

Baseline rMTs in the trained (*M* = 41.30, SE = 2.13) and untrained (*M* = 40.05, SE = 2.00) hemispheres of young adults did not differ from baseline rMTs in the trained (*M* = 40.05, SE = 1.70) or untrained (*M* = 41.35, SE = 1.53) hemispheres of older adults in the simple condition. ANOVA failed to reveal any differences in rMT between the hemispheres or between age groups (all *p’s* > 0.144).

**FIGURE 4:**
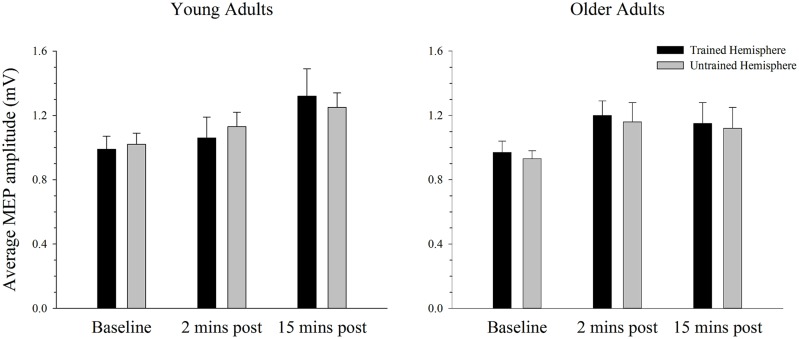
**Mean MEP amplitude before and after training on a simple motor task in young and older adults.** Average MEP amplitude increased from pre to post-training in both young and older adults, with the largest increase at the 15 min time-point (*p* < 0.05). There was no difference in the MEP increase between hemispheres or the two age groups. Error bars denote SEM.

The average MEP amplitude in the trained and the untrained hemispheres in young and older adults before and after training on the simple task is shown in **Figure 4**. It can be seen that MEP amplitudes increased significantly immediately after training, with a further increase at the second post-training interval in the young. ANOVA confirmed that training was associated with an increase in MEPs, as revealed by a significant main effect of time; *F*(2,76) = 7.66, *p* = 0.001, $\eta_{p}^{2}$ = 0.17. Paired samples *t*-tests revealed that MEP amplitude increased significantly from baseline to the 15 min time point [*t*(39) = 3.59, *p* = 0.001], with no difference between the two post-training measures [*t*(39) = 1.38, *p* = 0.177]. Compared to baseline there was a strong trend toward an increase at the 2 min post-training interval [*t*(39) = 2.46, *p* = 0.019; adjusted alpha level = 0.017]. There were no other significant main effects or interactions (all *p’s* > 0.166).

### Behavioral Performance in the Complex Task

**FIGURE 5:**
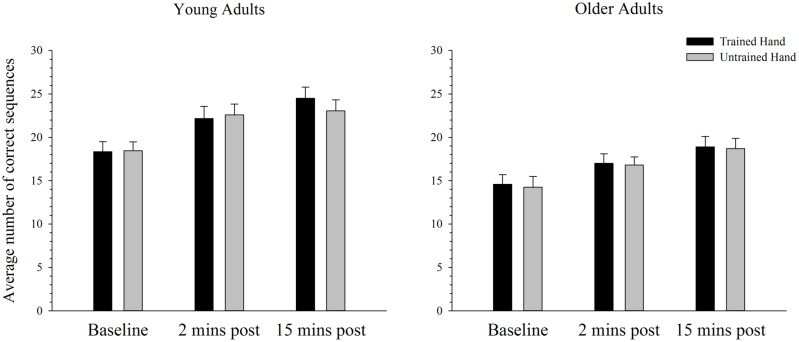
**Mean correct sequences completed before and after training on a complex motor task in young and older adults.** The average number of correct sequences completed increased significantly from baseline to both the 2 and 15 min post time-point in young and older adults in both the trained and the untrained hand (*p* < 0.05). Overall older adults completed fewer sequences than the younger group. Error bars denote SEM.

**Figure 5** depicts the average number of correct sequences performed with the trained and untrained hands of young and older adults before and after training on the complex task. As can be seen, the average number of correct sequences completed in the allotted time period increased significantly over time in both the trained and the untrained hands. ANOVA confirmed that performance improved after training with a significant main effect of time; *F*(2,76) = 102.42, *p* < 0.001, $\eta_{p}^{2}$ = 0.73. Paired samples *t*-tests revealed that performance significantly increased from baseline to both the two [*t*(39) = 8.70, *p* < 0.001] and 15 min [*t*(39) = 12.52, *p* < 0.001] post-training time points. A significant increase in performance was also evident from the 2 min to 15 min post-training measure; *t*(39) = 5.68, *p* < 0.001. As illustrated in **Figure 5**, overall performance also varied as a function of age, whereby young adults completed significantly more correct sequences than older adults. ANOVA supported this observation with a significant main effect of age: *F*(1,38) = 9.70, *p* = 0.003, $\eta_{p}^{2}$ = 0.20. There were no other significant main effects or interactions (all *p’s* > 0.100).

### Changes in Cortical Excitability in the Complex Task

**FIGURE 6:**
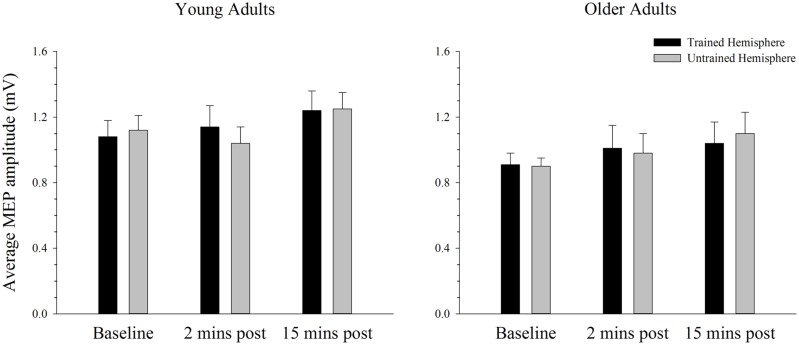
**Mean MEP amplitude before and after training on a complex motor task in young and older adults.** Average MEP amplitude trended toward an increase following training, with the greatest increase evident at the 15-min post-training time point. There were no differences across hemispheres or age groups. Error bars denote SEM.

Baseline rMTs in the trained (*M* = 40.40, SE = 2.08) and untrained (*M* = 40.30, SE = 1.76) hemispheres of young adults did not differ from baseline rMTs in the trained (*M* = 40.35, SE = 1.39) or untrained (*M* = 41.60, SE = 1.55) hemispheres of older adults in the complex condition (all *p’s* > 0.450). The average MEP amplitude in the trained and the untrained hemispheres in young and older adults before and after training on the complex task is shown in **Figure 6**. Overall MEP amplitude increased over time in the complex condition, with the largest increase emerging at the 15-min post-training time point. However, ANOVA revealed only a trend toward larger MEPs post-training with a marginal main effect of time; *F*(2,76) = 2.92, *p* = 0.062, $\eta_{p}^{2}$ = 0.07. Although the greatest increase was evident at the 15 min time point, paired sampled *t*-tests revealed that this increase was not significant [*t*(39) = 2.29, *p* = 0.028, adjusted alpha level = 0.017]. The increase evident at 2 min post-training [*t*(39) = 0.50, *p* = 0.620] and between the two post-training measures [*t*(39) = 1.99, *p* = 0.054] was also not significant. These effects did not differ between young and older adults or between the trained and untrained hemisphere, as indicated by the absence of any other significant main effects or interactions (all *p’s* > 0.644).

### Relationship between Cortical Excitability in the Untrained Hemisphere to Intermanual Transfer

**FIGURE 7:**
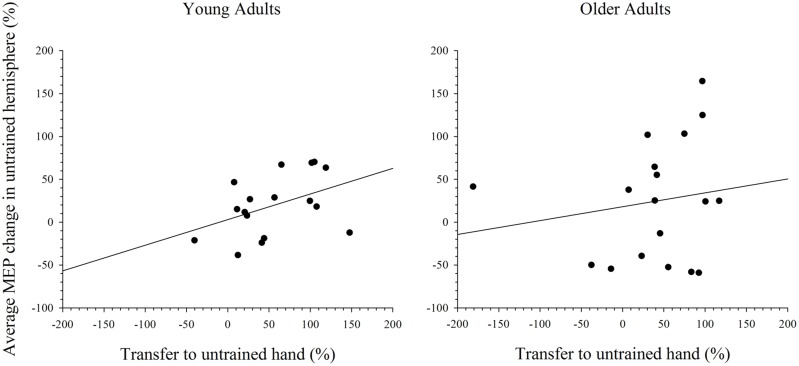
**Correlation between transfer of behavioral performance gains to the untrained hand and MEP change in the untrained hemisphere after training on the simple task.** Although there was a statistical trend toward an association between greater transfer and larger MEP change in the untrained hemisphere in young adults, this was not the case in older adults.

**FIGURE 8:**
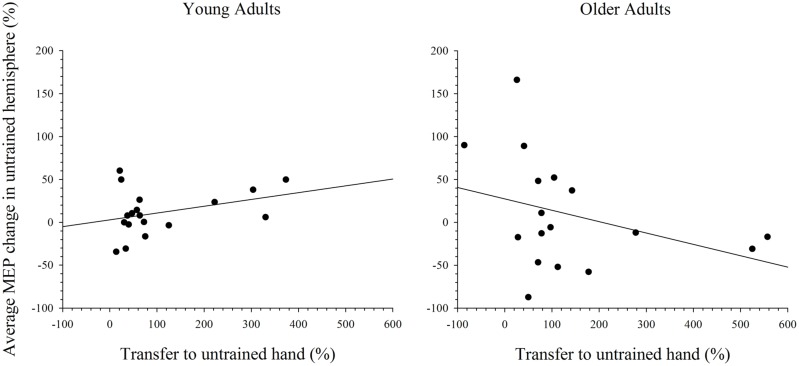
**Correlation between transfer of behavioral performance gains to the untrained hand and MEP change in the untrained hemisphere after training on the complex task.** Although greater transfer was non-significantly associated with greater MEP change in the untrained hemisphere in young adults, this relationship was reversed in older adults.

To further explore the notion that excitability changes in the two hemispheres differentially support intermanual transfer in young and older adults an index of transfer was created. This index represents the degree of performance gain in the untrained hand as a percentage of the performance improvement in the trained hand. Because the increase in MEP amplitudes were more reliable at the 15 min post-training interval, in both the simple and complex conditions, only data for this time point was included in the analysis. Data with Cook’s distance and studentized deleted residual values above 0.2 (i.e., Cook’s D > 4/*n*) and 2.0, respectively, were removed form the analyses; a maximum of three data points were removed from any analysis. As can been seen in **Figure 7**, higher scores on the transfer index were associated with greater MEP change in the untrained hemisphere of young adults. However, this positive correlation was only marginally reliable: *r*(16) = 0.428, *p* = 0.087. For older adults, there was also no reliable relationship between MEP amplitude change and intermanual transfer; *r*(17) = 0.162, *p* = 0.521. Data for the complex task are presented in **Figure 8**, which shows a positive relationship between the transfer index and MEP change in young adults, but a negative relationship in the older group. Analysis revealed, however, that there was no significant correlation between scores on the transfer index and MEP change in the untrained hemisphere in young [*r*(17) = 0.349, *p* = 0.156] or older [*r*(18) = -0.349, *p* = 0.170] adults.

## Discussion

The current study aimed to investigate whether intermanual transfer and bilateral cortical excitability are altered in older adults after motor training on simple and complex tasks. Both young and older adults demonstrated intermanual transfer following training with the dominant hand on a *simple* task, but overall older adults displayed lower levels of performance gains. Cortical excitability also increased comparably across the trained and untrained hemispheres in both young and old adults following training on the simple task. After training on a *complex* task, intermanual transfer was also evident in the young and older adults, but cortical excitability did not change in either hemisphere.

### Intermanual Transfer of a Simple Motor Skill is Maintained in Older Adults

Previous studies have demonstrated that intermanual transfer is reduced, or even absent in older adults (Hinder et al., 2011; Parikh and Cole, 2013), and that this effect is accompanied by bilateral increases in cortical excitability (Hinder et al., 2011). This observation suggests that older adults draw upon plastic changes in more diffuse brain regions (i.e., the homologous M1) to support learning of a simple motor task with the dominant hand. In the current study, however, intermanual transfer was observed in older adults. This effect is inconsistent with that found by Hinder et al. (2011) and Parikh and Cole (2013), but transfer has been reported when older adults trained on a simple task with their non-dominant hand (Hinder et al., 2013). There are two key methodological differences between the current study and the previous literature that may have contributed to the discrepant findings. First, in order to maximize kinesthetic similarity between simple and complex tasks the current study focused on the APB. Previous research, however, has focused solely on the first dorsal interroseous muscle (FDI). Although it is possible that intermanual transfer becomes increasingly muscle specific with age, the findings of Hinder et al. (2013), which demonstrate evidence of transfer in older adults with the FDI muscle suggest this is an unlikely explanation for the discrepancy between the findings of the current study and of Hinder et al. (2011). Second, in the current study the frequency and distribution of rest breaks given during training was reduced. Hinder et al. (2011) administered a 30 s break after every 10 trials during training, as well as a 5-min break mid-way through (after 150 trials). The current study, however, administered 30 s breaks only after every 30 trials and participants continued in this manner until all 300 movements were completed. The reduction in rest frequency may have played a role in maintaining intermanual transfer in the older adults by allowing greater time within a block to process and respond to feedback, and to encode and refine movement kinetics for maximum acceleration. However, Hinder et al. (2013) implemented the same rest periods as Hinder et al. (2011), and found evidence of intermanual transfer following training with the non-dominant hand in older adults. This suggests that neither alterations in the frequency of rest breaks nor hand dominance solely determine whether intermanual transfer manifests in older adults.

In addition to the methodological difference mentioned above, variation in sample characteristics could account for the divergent findings across studies. For example, particular lifestyle characteristics, educational experience, and occupational exposure can protect against age-related decline in cognition and memory. These factors can lead to differences in structural and functional neural networks or cognitive processes that allow some individuals to cope better with brain pathology, a process termed cognitive reserve (see Stern, 2009 for review). In addition, numerous factors, such as genetic polymorphisms (BDNF Val66Met; Cheeran et al., 2008), physical activity (Cirillo et al., 2009), and mirror activity (Hinder et al., 2011) have been shown to influence plasticity. Importantly, there is no reason to expect systematic differences in sample characteristics between the current study and those showing an absence of transfer effects, as all employed standard eligibility requirements for TMS (e.g., no neurological or psychiatric condition, not taking neuroactive medications) and similar recruitment methods. However, such differences cannot be ruled out. Future studies might attempt to survey a range of cognitive and lifestyle factors to determine which are associated with maintenance of transfer. Finally, it should be noted that mirror activity, which refers to the spillover of activity that can occur from an active limb to homologous muscles of a resting limb during unilateral movement, does not readily explain the transfer effects. Specifically, although mirror activity is more common in older adults it has been associated with reduced plasticity effects (Hinder et al., 2011), and thus does not explain the comparable bilateral plasticity evident in young and older adults in the current study.

The finding that intermanual transfer was evident in the presence of bilateral increases in cortical excitability in the current study does not support the idea that more diffuse plasticity is needed to support learning of the trained hand in older adults. Instead, the results could be taken to suggest that cortical excitability change in each hemisphere supported learning with the contralateral hand, as demonstrated in young adults by Lee et al. (2010). It is important to note, however, that MEP amplitude reflects the excitability of the corticospinal pathway, and that changes in spinal excitability were not examined in the current study. Nonetheless, evidence demonstrates that after motor training with the upper and lower limbs spinal excitability is not altered (Lagerquist et al., 2006; Mcdonnell and Ridding, 2006; Beck et al., 2007; Szecsi and Straube, 2007; Cirillo et al., 2010), suggesting that changes in MEP amplitude in the current study were driven predominately by cortical plasticity. But it remains possible that plasticity in the spinal cord in older adults may compensate for reductions in cortical plasticity. Evidence of reduced spinal plasticity in older adults (see Papegaaij et al., 2014 for review), however, argues against this possibility. As behavioral and MEP effects were similar across the post-measures, future studies might consider limiting MEP and behavioral measurement to a single time point. This would make room for the inclusion of paired pulse intracortical stimulation, which would aid in distinguishing pure cortical effects from changes at the spinal level (see Ziemann and Rothwell, 2000 for review).

In order to investigate further the relationship between cortical excitability change in the untrained hemisphere and transfer to the untrained hand correlations analyses were undertaken. The results of that analysis revealed that the relationship between greater excitability change in the untrained hemisphere and greater transfer of performance gains to the untrained hand was stronger in young, relative to older, adults. This finding lends support to the argument that the role of activity (plasticity) in the untrained hemisphere in supporting learning for the trained and untrained hands differs between young and older adults. Future studies would benefit from probing the casual relationship between activity in the untrained hemisphere and transfer to the untrained hand using repetitive TMS (rTMS). Specifically, studies similar to that of Lee et al. (2010), in which rTMS was used to interfere with activity in M1 immediately following a training task, would assist in identifying the differential role of activity within the untrained M1 for supporting learning with the trained and untrained hands in older adults.

### Performance Gains after Training on a Simple Motor Task are Reduced in Older Adults

Following training on the simple task, performance gains overall were lower in older relative to younger adults. At baseline, however, performance on the simple task was no different between young and older adults, suggesting that the reduction in performance gains in older adults was not due to an overall (basal) decrease in motor functioning. It is unlikely that this effect can be explained by muscle fatigue or a lack of motivation as participants were given frequent breaks during training and encouragement and feedback throughout the session. Instead, the reduction in training-related performance gains might be due to a capacity limit within the peripheral musculature, downstream of the motor cortex. Comparable cortical excitability changes between young and older adults following training supports this view. In this context there is evidence to suggest an overall decline in the musculature in older adults (Rice and Cunningham, 2002; Klass et al., 2008); remodeling of muscle fibers (Larsson, 2003), reduced motor neuron numbers (Doherty et al., 1993), and/or reduced motor unit numbers and activity (Doherty et al., 1993; Klein et al., 2001; Gordon et al., 2004) are just some age-related changes in musculature that might have contributed to reduced performance gains in older adults. Importantly, although the older adults showed an overall smaller increase in performance following training, there was no difference between the groups in the degree of intermanual transfer.

### Intermanual Transfer of Complex Motor Skills is Maintained in Older Adults

The current study demonstrates that although overall performance was lower in older relative to younger adults, intermanual transfer of a complex task was maintained in older adults. This result is consistent with the findings of Parikh and Cole (2013) who reported significant transfer in young and older adults after training on a grip and lift task with the dominant hand. Cortical excitability, however, did not change significantly in either hemisphere of young or older adults in the current study. Training on a complex task has been shown to increase excitability in M1 contralateral to the trained hand in young and older adults (Cirillo et al., 2011). However, there is a wealth of evidence suggesting that regions outside M1, such as pre-motor and supplementary motor regions are predominantly involved in performance and learning of complex sequential motor tasks (Sadato et al., 1996; Mima et al., 1999; Ehrsson et al., 2000, 2001; Solodkin et al., 2001; Verstynen et al., 2005; Davare et al., 2010; Holmström et al., 2011). The lack of plasticity in either M1 in young adults, at least as assessed with MEPs, is consistent with this evidence and further suggests that learning-related plasticity for complex sequential tasks occurs outside the primary motor cortices.

Due to age-related over-activity in diffuse brain regions, which was hypothesized to support learning of the motoric components of the task in the trained hand, older adults were predicted to show a greater increase in excitability in the untrained hemisphere after training on a complex task. This was not the case, as cortical excitability in young and older adults did not change from baseline after training on the complex task. Moreover, the presence of intermanual transfer in older participants following training of the simple task argues against over-activity in these individuals. Although these factors make it difficult to investigate the role of the untrained hemisphere in supporting learning with the trained hand in the current study, correlations between MEP change in the untrained hemisphere and transfer of behavioral gains to the untrained hand after training on the complex task were assessed. The results reveal a negative relationship in older adults whereby greater change in MEP amplitude in the untrained hemisphere was weakly associated with a decrease in transfer to the untrained hand. In young adults, however, a positive relationship was evident. This suggests that the increase in cortical excitability in the untrained hemisphere may reflect the increased role of the untrained hemisphere in supporting learning with the trained hand in older adults.

Future studies would benefit from using a combination of imaging techniques and TMS to probe bilateral training-related plasticity, to identify the networks contributing to learning and transfer of complex motor skills and the extent to which those networks are altered in the aged brain. As discussed previously, an important node in the motor network that has been shown to be involved in learning complex tasks is the premotor cortex (Mima et al., 1999). In addition to identifying the degree of involvement of this region in young and older adults during the performance of complex tasks with imaging techniques, excitatory or inhibitory repetitive TMS to this region could help to further establish the causative contribution of premotor regions in learning complex tasks in young and older participants. Further, future studies might also use twin coil TMS to probe intracortical inhibition between premotor and primary motor cortex prior to and following a training intervention to investigate training related change in the connections between these regions and how these might be altered by advancing age.

## Conclusion

The current study provides evidence to suggest that intermanual transfer of both simple and complex tasks is maintained in older adults. The findings of the current study also suggest, however, that the extent to which cortical excitability of the untrained hemisphere supports transfer of performance gains to the untrained limb may differ in young and older adults. Future studies that interfere with activity in the motor cortices immediately following training and measure the impact of such interference on behavioral performance would help to establish the role of the untrained hemisphere in supporting intermanual transfer in older adults. Nonetheless, the current study carries practical implications for rehabilitation practices involving intermanual transfer effects. For example, it is possible that individuals experiencing limb deficits (e.g., following stroke) may benefit from training with the intact hand. However, it is important to remember that in pathological conditions, such as stroke, cortical activity can be significantly altered (Nowak et al., 2009), which could interact with the training-related changes we describe here. Finally, based on the current results it would be predicted that techniques implementing such training tasks are likely to benefit both young and older adults similarly.

## Author Contributions

All authors were substantially involved in the conception and design of the study, in the interpretation of data and in revision of the paper. DD acquired and analyzed the data and wrote the manuscript.

## Conflict of Interest Statement

The authors declare that the research was conducted in the absence of any commercial or financial relationships that could be construed as a potential conflict of interest.
